# Jules Emile Pean

**Published:** 1898-04

**Authors:** 


Jules Emile Pean, the eminent French surgeon, is dead. M. Pean devised the artery catch-forceps that bear his name and left a surgical fame that will last for centuries. He died of pneumonia January 30, 1898, at the age of 67 years, having practised surgery continuously at Paris for more than 45 years. His funeral oration was pronounced by Dr. Samuel Pozzi, the eminent gynecologist, who has lately been chosen life senator.

